# *PIK3CA* and *PTEN* Genes Expressions in Breast Cancer

**DOI:** 10.31557/APJCP.2019.20.9.2841

**Published:** 2019

**Authors:** Nabil Hadi Alowiri, Shaden Muawia Hanafy, Reham Abdel Haleem, Ahmed Abdellatif

**Affiliations:** 1 *Department of Molecular Biology, Genetic Engineering and Biotechnology Research Institute, University of Sadat City, Sadat City,*; 2 *Department of Clinical Pathology,*; 3 *Surgical Oncology Unit, Department of General Surgery, Faculty of Medicine, Alexandria University, Alexandria, Egypt. *

**Keywords:** PIK3CA, PTEN, expressions, breast cancer

## Abstract

**Background:**

The *phosphatidylinositol-3 kinase (PI3K)* intracellular signaling pathway plays an important role in breast cancer. The current study aimed to evaluate the expressions of two main regulators of PI3K pathway; phosphatidylinositol-3- kinase catalytic subunit alpha as activator (*PIK3CA)*, and phosphatase and tensin-homolog as inhibitor *(PTEN)*, in breast carcinoma tissue, and compare with their expressions in adjacent normal breast tissue.

**Methods:**

A total of fifty female patients with breast carcinoma from surgical oncology unit of Alexandria-Main University Hospital were included in this study. The Quantitative Real Time PCR was used to quantify expressions of *PIK3CA* and *PTEN*.

**Results:**

*PIK3CA mRNA* expression was significantly increased in breast cancer tissues compared to normal breast tissues (P<0.001, Z=5.700), also *PTEN mRNA* expression was significantly higher in breast carcinoma tissue compared to normal breast tissue (P<0.001, Z=5.362).

**Conclusion:**

Increased the expressions of *PIK3CA* and *PTEN mRNA* in breast cancer tissue compared to normal breast tissue.

## Introduction

Breast cancer (BC) is a major lethal public health problem in women worldwide, it is the second leading cause of cancer death after lung cancer in women with more than one million new cases are diagnosed every year (Siegel et al., 2016; Segal et al., 2018). In 2018, one in eight women in the USA had a lifetime risk of developing breast cancer (Segal et al., 2018), while in Egypt according to a previous a study the breast cancer accounts for 15.4% of all cases of cancer but in female it accounts for 32.04% of female cases of cancer (Ibrahim et al., 2014). The molecular biomarkers, and their correlation with pathological parameters are very important for diagnosis, prognosis, predictive utility, management, and prevention of breast cancer (Patani et al., 2013).

The *phosphatidylinositol-3-kinase (PI3K)* pathway is an important signaling pathway in cells and is involved in essential cellular functions such as metabolism, proliferation, survival, motility, and growth and plays a main role in development and progression of breast cancer (Engelman et al., 2006; Boyault et al., 2012).

Based on structure, regulation, and substrate specificity, the PI3K family is classified into three classes, class I, class II, and class III (Leevers et al., 1999). Class I is divided to IA and IB, class IA PI3K is composed of two subunits: A regulatory subunit (85 kDa), and a catalytic subunit (110 kDa) of which contain four isoforms (α, β, γ, and δ) that can be activated by tyrosine kinase receptors (Bellacosa et al., 2005). The α isoform of catalytic subunit (*PIK3CA*) is encoded by *PIK3CA* gene which is located on chromosome 3 (3q26.32). It has been found amplified or mutated in several cancer types including breast, ovary, colon, liver, stomach, brain, and lung (Samuels et al., 2004; Levine et al., 2005). When PI3K activated converts the phosphatidylinositol -4,5-bisphosphate (PIP2) to phosphatidylinositol -3,4,5-trisphosphate (PIP3) that leads to downstream signaling through a PI3K/AKT/mTOR pathway (Wang et al., 2011).

On the other hand, the PI3K activation is inhibited by phosphatase and tensin-homolog (PTEN) which converts PIP3 to PIP2 (Wang et al., 2011). PTEN protein is involved in DNA repair, apoptosis, cell cycle progression, and proliferation, and is encoded by PTEN gene, it is a tumor suppressor gene located on chromosome 10 (10q23). Changes of expressions is the most common alteration of PTEN that may leads to tumor cell growth, escape from cell cycle arrest, and apoptosis signals (Leslie and Downes 2004; Song et al., 2012). 

The PI3K/AKT/mTOR pathway has a major role in the resistance to anticancer drugs and in the response to treatment (Leslie and Downes, 2004). In the recent strategies of cancer treatment and depending on PI3K pathway can be use the PI3K inhibitors to cancer treatment which is used alone or combined with other strategies of cancer therapy (Fruman and Rommel, 2014, Arnedos et al. 2015).

The present study aimed to assess the mRNA expression of *PIK3CA* and PTEN genes in the breast carcinoma tissue compared to their expression in the normal breast tissue, and evaluated the correlation of their expression with clinicopathological parameters in patients with BC.

## Materials and Methods

*Patients and samples*

The current study was conducted on fifty female patients with breast cancer selected from surgical oncology unit of Alexandria-Main University Hospital, Alexandria, Egypt. The study was previously approved by the Ethical Committee of the Genetic Engineering and Biotechnology Research Institute, University of Sadat City. Fresh tissue of breast carcinoma and adjacent normal tissue were collected from each patient during the excision surgery and immediately stored at -80°C. Discrimination between breast cancer tissues and the adjacent normal tissues was made by histopathological examinations.

Clinical parameters were collected for all patients, including age, tumor size, type, grade, lymph node metastasis, and immunohistochemical situation of estrogen receptor (ER), progesterone receptor (PR), and human epidermal growth factor receptor 2 (HER-2).

*RNA extraction and cDNA synthesis*

The RNA was extracted using PureLink^®^ RNA Mini Kits for purification of total RNA (Invitrogen ™, Carlsbad, CA 92008, USA). Catalog Number (12183018A) according to the manufacture instructions. Fifty mg of frozen tissue were used to extract the total RNA, the tissues were homogenized and grinded by a Tissue Lyser LT (Qiagen, Germany). It was operated for 5 min at 50 Hertz. The quantity and purity of extracted RNA was determined by NanoDrop ND-2000/2000c spectrophotometer (ThermoScientific, USA).

High-Capacity cDNA Reverse Transcription Kit was used for cDNA synthesis (Applied Biosystems, Carlsbad, CA 92008, USA) according to the manufacture instructions.

*Quantitative expression examination*

Quantitative Real Time PCR (qPCR) was used to determine the expression levels of *PIK3CA* and PTEN genes. GAPDH was used as endogenous reference for both genes. The relative expression was determined using Applied Biosystem 7500 Fast Dx Real- Time PCR System (Applied Biosystem, USA).

GAPDH, as the appropriate housekeeping gene, was used to control for variations in RNA concentration and integrity. The qPCR was performed using the Maxima SYBR Green qPCR Master Mix (2X) kit (Ferments, Thermo Fisher Scientific Inc. Catalog Number K0251). Specific primers were used to quantify* PIK3CA*, *PTEN*, and *GADPH* genes (Ferments, Thermo Fisher Scientific Inc. USA). The sequences of primers were: *PIK3CA* Forward: GGCCACTGTGGTTGAATTGGGA, Reverse: AGTGCAC-CTTTCAAGCCGCC, *PTEN *Forward: TGGGCCCTGTACCATCCCAAGT, Reverse: TGTGGCAACCACAGCCATCGT, GAPDH Forward: AAGGTCGGAGTCAACGG-ATTTG, and Reverse: GCCATGGGTGGAATCATATTGG. The amplicons size of the amplified products were: *PIK3CA 250 pb*, *PTEN 445 pb*, and* GAPDH 150 pb*. Real-Time PCR System was programmed as follows: one cycle at 95ºC for 10 min, 40 cycles at 95ºC for 15 sec, and 60ºC for 60 sec. Melting curve analysis was used to determine the specificity of the PCR products after each run.

*Statistical analysis*

Data were fed to the computer and analyzed using IBM SPSS software package version 20.0. (Armonk, NY: IBM Corp) Qualitative data were described using number and percent. Quantitative data were described using range (minimum and maximum), mean, standard deviation and median. The t-test (t), chi-square test (χ^2^), Wilcoxon signed ranks test (Z), and Spearman coefficient (rs) were performed for statistical examination.

## Results

The present study included fifty female patients with breast cancer, and they had a mean age 54 age years (range: 19-80 years). The clinicopathological features of all patients were listed in Table 2. 

**Figure 1:**
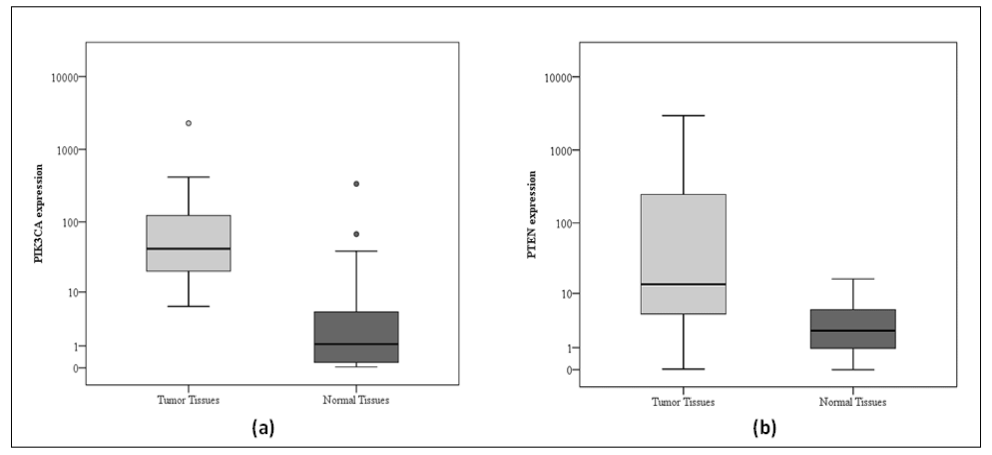
Descriptive Analysis of the Studied Cases According to *PIK3CA* (a) and *PTEN* (b) Expressions (n = 50)

 When we compared *PIK3CA mRNA* expression in breast carcinoma tissues with its expression in normal breast tissues observed significantly higher expression in carcinoma tissues than in normal tissues (P<0.001, Z=5.700). The median value for *PIK3CA/GADPH* ratio in breast carcinoma tissues was 42.35 compared to 1.12 *PIK3CA/GADPH* in normal breast tissues. High *PIK3CA* expression (above median level 42.35) was in 25 patients (50%), while the low *PIK3CA* expression (≤42.35) was in 50% of patients (25 patients). In breast tumor tissue observed increase the *PIK3CA mRNA* expression in 47 patients out of 50 (94%). Table1, Figure 1a.

**Table 1.** Descriptive Analysis of the Studied Cases According to *PIK3CA* and *PTEN* Expression (n = 50)

	Tumor Tissues	Normal Tissues	Z	p
*PIK3CA* expression			5.700*	<0.001*
Min. - Max.	5.98 – 2288.2	0.03 – 335.5		
Mean ± SD.	131.4 ± 328.6	11.45 ± 48.12		
Median	42.35	1.12		
*PTEN* expression				
Min. - Max.	0.02 – 2957.2	0.0 – 16.34		
Mean ± SD.	281.3 ± 554.7	4.16 ± 4.32	5.362*	<0.001*
Median	13.76	2.42		

Z, p, p value for Wilcoxon signed ranks test for comparing between pathogenic and normal; *, Statistically significant at p ≤ 0.05

**Table 2:** Relations between *PIK3CA* and *PTEN* Genes Expressions and Clinicopathological Features of BC

	*PIK3CA* expression (50)		significance		*PTEN* expression (50)		significance	
	Low *PIK3CA*	High *PIK3CA*	X^2^	P value	Low *PTEN*	High *PTEN*	X^2^	P value
Number, N (%)	25 (50%)	25 (50%)			25 (50%)	25 (50%)		
Age, median (range)	53 (19-72)	55 (36-80)		0.421	51 (19-73)	55 (35-80)		0.16
Tumor size, mm, median (range)	17 (7.8-18)	19 (8.3–20.4)		0.291	17 (9.6-40)	19 (5-33)		0.289
Histology, N (%)								
Ductal	23 (92)	22 (88)	0.222	1	23 (92)	22 (88)	0.222	1
Lobular	2 (8)	3 (12)			2 (8)	3 (12)		
Tumor grade, N (%)								
I	4 (16)	2 (8)			1 (4)	5 (20)		
II	9 (36)	16 (64)	3.864	0.147	14 (56)	11 (44)	2.924	0.275
III	12 (48)	7 (28)			10 (36)	9 (36)		
ER status, N (%)								
Positive	7 (28)	8(32)	0.095	0.758	6 (24)	9 (36)	0.857	0.355
Negative	18 (72)	17 (68)			19 (76)	16 (64)		
PR status, N (%)								
Positive	11 (44)	14 (56)	0.72	0.396	10 (40)	15 (60)	2	0.157
Negative	14 (56)	11 (44)			15 (60)	10 (40)		
HER2 status, N (%)								
1+	11 (44)	10 (40)			9 (36)	12 (48)		
2+	7 (28)	7 (28)	0.114	0.944	9 (36)	5 (20)	1.638	0.441
3+	7 (28)	8 (32)			7 (28)	8 (320		
Lymph node status, N (%)								
Metastasis	6 (24)	8 (32)	0.397	0.529	8 (32)	6 (24)	0.397	0.529
No metastasis	19 (76)	17 (68)			17 (68)	19 (76)		

High PIKECA expression >42.35 (median); low PIKECA expression ≤42.35; High PTEN expression >13.76 (median); low PTEN expression ≤13.76.

**Table 3:** Correlation between *PIK3CA* Expression and *PTEN* Expression in Breast Carcinoma

*PTEN* expression	*PIK3CA* expression	
	r_s_	p
	-0.051	0.723

r_s_, Spearman coefficient

Also, *PTEN mRNA* expression was significantly higher in carcinoma tissues than in normal breast tissues (P<0.001, Z=5.362). The median value for *PTEN/GADPH* ratio in breast carcinoma tissues was 13.76 compared to 2.42 *PTEN/GADPH* in normal breast tissues. High *PTEN *expression (above median level 13.76) was in 25 patients (50%), and the low *PTEN* expression (≤13.76) was in 25 patients (50%). In the *PTEN* gene, only 8 (16%) out of 50 carcinoma samples had decreased gene expression, while the 42 (84%) had increased expression. Table 1, Figure 1b.

Clinicopathological characteristics did not show any significant association with *PIK3CA* and* PTEN* genes expressions (P>0.05), Table 2. 

*PIK3CA* expression in breast carcinoma did not show a significantly positive correlation with *PTEN* expression (rs= -0.05, P=0.723), Table 3.

## Discussion

Breast cancer is the most common cancer among women, and the second most frequent cancer worldwide (Siegel et al., 2016; Segal et al., 2018). *PI3K* pathway play a crucial role in development and progression of cancers mostly breast cancer (Boyault et al., 2012), in this study we focused on the main regulators of the *PI3K* pathway (*PIK3CA *which acts as *PI3K* activator and *PTEN* acts as *PI3K* inhibitor and tumor suppressor), and were examined to test whether an increased* PIK3CA *expression and decreased *PTEN* expression in breast carcinoma and normal breast tissue.

Most previous studies to assess genes expressions have used immunohistochemistry (IHC) methods that are based on detect proteins molecules by antibodies, (Tsutsui et al., 2005; Aleskandarany et al., 2010) and there were some studies used quantitative method by real time PCR to assess the mRNA expressions of target genes. In this study used also the real time qPCR to detect the mRNA expressions of *PIK3CA* and *PTEN *genes because this method is a highly sensitive, quantitative, reproducible and accurate (Vanden et al., 2005; Salmani et al., 2018).

In the current study, we measured the mRNA expression of *PIK3CA* and *PTEN* genes in breast cancer tissue and compared them with their expression in normal breast tissue, and according to data of this study the *PIKECA* expression showed significantly higher in breast carcinoma tissue than in normal breast tissue, where the median value of* PIK3CA *expression in breast carcinoma was 42.35 whereas in normal tissue was 1.12 (P<0.001). This result may support the hypothesis of the PI3K pathway play a crucial role in development and progression of breast cancer (Boyault et al., 2012).

To our knowledge, there is not many studies about *PIK3CA* gene expression in breast cancer, most studies focused on *PIK3CA* mutations in breast cancer.* PIK3CA *expression data in the current study similar to data in the Palimaru et al., (2013) study that showed significantly higher *PIK3CA* expression in breast carcinoma than in normal breast tissue (p=2x10^-11^), *PIK3CA* expression was increased in breast carcinoma in 76% of patients (114/149) whereas in our study the *PIK3CA* expression was increased in breast carcinoma in 96% of patients (47/50). Also, there was a previous report at 2010 (Aleskandarany et al., 2010) showed *PIK3CA* high expression in breast tumor tissue with IHC.

We compared the *PIK3CA mRNA* expression with clinicopathological parameters (age, tumor size, tumor histology, tumor grade, *ER*, *PR*, *HER2*, and lymph node status) and we did not observe significant association between *PIK3CA* expression with any clinicopathological parameter (P>0.05), that is corresponds to the results of the Palimaru et al. study (Palimarun et al., 2013), and conflict with Aleskandarany et al., (2010) study, which reported significant associated between increased *PIK3CA* expression with histologic tumor type, higher tumor grade, larger tumor size, negative hormone receptors (*AR* and *PR*), positive *HER2*, and lymph node metastases, and showed no significant association with age.

The *PTEN* gene encodes a lipid phosphatase that acts as an inhibitor of *PI3K* and influences on the *AKT* pathway regulations. Decreased or loss of *PTEN* expression leads to activated and increased levels of the *AKT*, thus promoting cell cycle progression, cell survival, proliferation, and migration, this is a major cause of tumors creation (Majumder and Sellers, 2005). The results of *PTEN* gene expression in this study were contrary to expected, the expression was significantly higher in carcinoma tissue than in normal breast tissue (P<0.001, Z=5.362) where the median value of *PTEN* expression in breast cancer tissue was 13.76 but in normal tissue was 2.42. The current study showed decreased in* PTEN* expression in breast tumor tissue in only 16% of patients compared to expression in normal breast tissue, which was similar to Palimaru et al study (Palimaru et al., 2013), it has been reported that low of* PTEN mRNA* expression in breast carcinoma tissue occurs in only 20% of patients compared to normal breast tissue. In a study at 2018 for measuring *PTEN mRNA* expression in breast carcinoma tissue and compared to expression in normal breast tissue for 78 breast cancer patients, a reduced *PTEN *expression in breast carcinoma tissue was found in only 33.3% of patients (Salmani et al., 2018). The two previous studies used quantitative method by real time PCR for assessment *PTEN mRNA* expression. Also, a reduced or absent of *PTEN* expression in breast cancer tissue was only in 28 out 85 of breast cancer patients (33%) in the Engin et al., (2006) study, which was using IHC for the detection of *PTEN*. There were other studies did not correspond to our data in this study, and reported decreased or absent *PTEN* expression in breast carcinoma tissue in high percentage among the breast cancer patients (Kechagioglou et al., 2014; Gschwantler-Kaulich et al., 2017; Shabbir et al., 2017).

As listed in Table 2, *PTEN* expression did not have any relation to any clinicopathological factors (P>0.05), there were also some studies that showed no association between *PTEN* expression and clinicopathological factors of breast cancer. The study mentioned above reported significance relation between* PTEN* expression and lymphatic invasion only (P=0.046), and showed not association with others clinicopathological parameters (Salmani et al., 2018). Also, the Palimaru et al., (2013) study did not find correlation between *PTEN* expression and lymph node status or with any clinicopathological factors, but other studies reported presence relation between *PTEN* expression and some clinicopathological factors (Li et al., 2015; Golmohammadi et al., 2016; Wang et al., 2017). A study at 2017 conducted a meta-analysis of 27 studies which included 10,231 patients to assess the relations of *PTEN* expression with clinicopathological characteristics, the *PTEN* loss had relations with larger tumor size (P=0.0006), lymph node metastasis (P=0.0001), negative ER (P=0.03), and negative PR (P=0.02), and reported no significant relation between *PTEN* loss and *HER2* status (Li et al., 2017).

*PIK3CA* plays important role in breast cancer treatment, the FDA on May 2019 approved alpelisib (Piqray tablets) to be used in combination with fulvestrant (endocrine therapy) for the treatment of postmenopausal women, and men, with *HR+*,* HER2-*, *PIK3CA*-mutated, advanced or metastatic breast cancer. The treatment decision by this drug depend on identification of *PIK3CA* mutations, therefore the FDA also approved the therascreen *PIK3CA* Kit (*PIK3CA* biomarkers) for detection of activating mutations in the *PIK3CA* gene (ASA, 2019). *PIK3CA* gene mutations are the most common in *HR+/HER2- BC*, which are estimated to be present in approximately 40% of *HR+/HER-* advanced or metastatic BC patients (Tolaney et al., 2019).

According to our results, we encourage and recommend further studies include a large size number of breast cancer patients to further evaluate the relations of *PIK3CA* and *PTEN* genes expressions to clinical pathological characteristics of breast cancer, and assess the* PIK3CA* and *PTEN* proteins expressions and compare with the mRNA expression of these genes.

In conclusion, the results of the current study showed higher significantly increased* PIK3CA mRNA* expression in breast tumor tissue compared to normal breast tissue, and contrary to expected, we also observed increased *PTEN* expression in breast carcinoma tissue compared to normal breast tissue. The expressions of *PIK3CA *and *PTEN* showed not significant relation with any clinicopathological characteristics of breast cancer, and reported not found correlation between* PIK3CA* and *PTEN* expressions.
